# Cutaneous Psoriasis and Symptoms (Itch, Pain, and Burning Sensation): A Monocentric Retrospective Study on 299 Patients in Italy

**DOI:** 10.3390/jcm14134388

**Published:** 2025-06-20

**Authors:** Lidia Sacchelli, Federica Filippi, Camilla Loi, Giacomo Clarizio, Tullio Brunetti, Michelangelo La Placa, Federico Bardazzi

**Affiliations:** 1 Dermatology Unit, IRCCS Azienda Ospedaliero-Universitaria di Bologna, 40138 Bologna, Italy; lidia.sacchelli@gmail.com (L.S.); federicafilippi8@gmail.com (F.F.); camilla.loi30@gmail.com (C.L.); tullio.brunetti@studio.unibo.it (T.B.); michelangelo.laplaca@unibo.it (M.L.P.); federico.bardazzi@aosp.bo.it (F.B.); 2 Department of Medical and Surgical Sciences, Alma Mater Studiorum University of Bologna, 40138 Bologna, Italy

**Keywords:** psoriasis, itch, burning sensation, pain, tingling

## Abstract

**Background:** Psoriasis is a chronic inflammatory skin disease with a strong psychosomatic component. While clinical severity is traditionally measured using the PASI and BSA, subjective symptoms such as itch, pain, and burning sensation significantly impact patients’ quality of life and remain under-assessed. **Methods:** We conducted a retrospective observational study on 299 adult patients with psoriasis evaluated at a tertiary dermatology center in Italy. Data on itch, pain, and burning were collected using validated patient-reported outcome measures. Disease severity (PASI and BSA) and quality of life (DLQI) were recorded. Associations between symptoms and clinical variables were statistically analyzed. **Results:** Itch was the most frequent symptom, reported by 73% of patients in the previous 4 weeks. Burning and pain were reported by 43% and 27%, respectively. Longer disease duration was associated with increased itch and burning (*p* < 0.05). Patients receiving systemic treatment showed significantly fewer symptoms (*p* < 0.05). Higher PASI and BSA scores correlated with a greater itch intensity. Importantly, significant symptoms were also reported by patients with low clinical severity. Higher DLQI scores were associated with increased symptom burden and emotional distress. **Conclusions:** Subjective symptoms such as itch, burning, and pain are frequent, clinically relevant, and not always proportional to visible disease severity. These findings underscore the need for routine symptom assessment in psoriasis and support a patient-centered approach in both clinical practice and therapeutic strategies.

## 1. Introduction

Psoriasis is a chronic, immune-mediated inflammatory skin disease with a global prevalence of approximately 2–4% [1]. It is characterized by erythematous, scaly plaques that can occur anywhere on the body but frequently affect the scalp, elbows, knees, and trunk. While traditionally considered a dermatological condition, psoriasis is now recognized as a systemic disease associated with multiple comorbidities, including psoriatic arthritis, cardiovascular disease, metabolic syndrome, and depression [2]. Over the last few decades, research and clinical management have focused predominantly on the visible burden of the disease—namely, the extent and severity of skin lesions—assessed using objective scoring tools such as the Psoriasis Area and Severity Index (PASI) and Body Surface Area (BSA) involvement.

However, psoriasis also entails a substantial subjective burden, often overlooked in clinical practice. Symptoms like pruritus, burning sensation, and skin pain are highly prevalent and significantly impair quality of life, despite not being directly proportional to visible disease severity [3]. These symptoms can affect patients’ sleep, concentration, emotional balance, sexual life, and ability to engage in social or professional settings [4,5]. Nevertheless, such symptoms are rarely the primary focus during routine clinical evaluations and are underrepresented in standard severity indices. The Dermatology Life Quality Index (DLQI), widely used to capture patient-reported outcomes, includes only one question specifically addressing skin symptoms, underscoring a historic lack of attention to this aspect of the disease experience [3].

In the early 2000s, Finlay et al. proposed the “Rule of Tens” as a practical guide to identify candidates for biologic treatments. According to this rule, patients with PASI ≥ 10, BSA ≥ 10%, or DLQI ≥ 10 were considered eligible for advanced systemic therapies [2]. This composite approach allowed clinicians to balance objective and subjective disease parameters. However, it still prioritized the extent and visibility of lesions over the lived experience of the patient. Notably, the DLQI’s limited symptom assessment meant that distressing symptoms like pruritus or pain could be underappreciated in treatment decision-making.

In recent years, there has been a paradigm shift in dermatological care toward a more patient-centered approach. Guidelines and consensus statements have emphasized the need to consider special site involvement (such as the scalp, face, genital area, hands, and feet) and the psychosocial impact of the disease when determining treatment eligibility [6]. These “high-impact” areas may involve relatively small surface areas but cause disproportionate distress due to their visibility, functional importance, or cultural sensitivity. In this context, subjective symptoms like itch or pain have emerged as additional key elements in the comprehensive assessment of disease burden.

Itch (pruritus) is now recognized as one of the most frequent and burdensome symptoms in psoriasis. Once thought to be a minor component—or even absent altogether; it is now estimated to affect up to 70–90% of psoriatic patients [1]. Burning sensations and cutaneous pain are also commonly reported, with prevalence rates ranging from 20% to 50%, depending on the population studied [7,8,9]. These symptoms are often interrelated and may coexist, further compounding patient discomfort. For example, pruritus and burning may alternate or overlap in patients with scalp or genital psoriasis, making symptom categorization and management even more challenging.

From a pathophysiological perspective, these symptoms are believed to result from neuroimmune interactions in the skin. Recent studies have identified a role for cytokines such as IL-17, IL-23, IL-31, and TNF-α in the sensitization of peripheral nerves, leading to aberrant activation of pruriceptors and nociceptors [10,11]. Additionally, increased expression of nerve growth factor (NGF) and altered densities of intraepidermal nerve fibers have been described in psoriatic plaques, suggesting that local neural remodeling may underlie some of the sensory abnormalities [4,5,7]. These findings support the view that psoriatic skin is not only inflamed but also functionally altered in terms of sensory perception.

Despite growing recognition of the importance of these symptoms, their incorporation into treatment algorithms remains inconsistent. While the latest guidelines acknowledge their relevance, quantifying symptoms and translating them into actionable thresholds for treatment escalation is still an evolving challenge. This underscores the need for studies that investigate the prevalence, intensity, and clinical correlates of itch, pain, and burning in psoriasis, particularly in real-world settings.

Furthermore, recent research has highlighted the close relationship between skin symptoms and mental health. Chronic itch and pain have been associated with increased rates of anxiety, depression, sleep disorders, and even suicidal ideation in psoriatic patients [12]. In this light, treating these symptoms is not only a matter of physical relief but also of psychological and psychiatric importance. A patient who may appear stable from a PASI or BSA perspective could still experience significant suffering due to persistent or fluctuating symptoms.

Given the evolving understanding of psoriasis as a systemic and psychosensory disease, it becomes essential to integrate the assessment of subjective symptoms into clinical practice and research. This includes using validated patient-reported outcome measures, adopting shared decision-making frameworks, and tailoring therapies to individual patient needs, not just visible lesions.

The primary objective of this study is to assess the prevalence and burden of pruritus, a burning sensation, and skin pain in a cohort of real-world patients with psoriasis. In addition, we aim to explore the correlation of these symptoms with clinical severity (PASI and BSA), quality of life (DLQI), and treatment modality. By doing so, we hope to contribute to a more nuanced and empathetic understanding of the psoriatic patient experience and to advocate for the inclusion of sensory symptoms in therapeutic decision-making.

## 2. Materials and Methods

This study is a retrospective observational analysis based on real-world data collected from psoriatic patients attending a tertiary referral center. The study population consisted of adult patients diagnosed with psoriasis who were evaluated at the Dermatology Unit of the Policlinico Sant’Orsola Malpighi in Bologna between 1 January and 31 May 2019. The primary aim was to evaluate the presence and intensity of subjective symptoms—specifically, pruritus, a burning sensation, and skin pain—and their relationship with disease severity and quality of life.

### 2.1. Inclusion Criteria

Participants were included in the study if they met all the following criteria:

- Age ≥ 18 years;
- Confirmed clinical diagnosis of psoriasis by a board-certified dermatologist;
- Provided written informed consent for the retrospective use of anonymized clinical data;
- Available documentation reporting the presence of pruritus, a burning sensation, or skin pain.

Patients with incomplete symptom data or unconfirmed psoriasis diagnoses were excluded. Only those with complete clinical records and symptom assessment questionnaires were analyzed.

### 2.2. Symptom Assessment

Subjective symptoms were evaluated using standardized and validated patient-reported outcome measures (PROMs). The severity of pruritus was quantified using a Visual Analog Scale (VAS), ranging from 0 (no itch) to 10 (worst imaginable itch). Patients were asked to score their itch intensity over the previous four weeks (Supplementary Table S1).

Burning sensation and skin pain were assessed using the Numeric Rating Scale (NRS), also ranging from 0 to 10 (Supplementary Tables S2 and S3).

These scales allowed patients to independently rate the intensity of each symptom without interference from clinical staff, ensuring consistency and minimizing reporting bias.

In addition to symptom scores, patients were asked to indicate the duration of symptoms, their distribution across body areas, and whether the sensations were constant or fluctuating. The questionnaires also included specific questions on the impact of symptoms on sleep quality, emotional well-being (such as anxiety or depressive symptoms), and concentration at work or during daily activities.

### 2.3. Assessment of Psoriasis Severity and Quality of Life

The clinical severity of psoriasis was evaluated by trained dermatologists using standard measures: Psoriasis Area and Severity Index (PASI) and Body Surface Area (BSA) involvement. These metrics were recorded on the same day as the symptom questionnaires were completed, ensuring that data reflected the same disease state.

Health-related quality of life was assessed using the Dermatology Life Quality Index (DLQI), a validated 10-item questionnaire with scores ranging from 0 (no impact) to 30 (maximum impact on quality of life). The DLQI scores were categorized into standard brackets: 0–1 (no effect), 2–5 (small effect), 6–10 (moderate effect), 11–20 (very large effect), and 21–30 (extremely large effect).

### 2.4. Statistical Analysis

Descriptive statistics, including frequencies, percentages, means, and standard deviations, were used to summarize patient demographics, symptom prevalence, and disease severity. Comparisons between subgroups—based on sex, age, treatment type, psoriasis subtype, and presence of comorbidities—were performed using chi-square tests for categorical variables and *t*-tests or ANOVA for continuous variables.

Pearson’s correlation coefficients were calculated to assess the relationships between symptom severity (VAS/NRS scores) and PASI or BSA scores. Linear regression models were used to evaluate predictors of DLQI scores, particularly examining whether subjective symptoms independently contributed to impaired quality of life beyond clinical severity scores. *p*-values < 0.05 were considered statistically significant.

Subgroup analyses were also conducted to explore symptom prevalence in specific psoriasis phenotypes, including palmoplantar, scalp, and genital involvement. The presence or absence of psoriatic arthritis (PsA) was evaluated as a potential modifier of symptom burden. Education level and occupation were also analyzed to assess whether sociodemographic factors influenced symptom perception or reporting.

### 2.5. Ethical Considerations

The study received ethical approval from the Ethical Committee of the Policlinico Sant’Orsola Malpighi (protocol code “PSO_SIN”). The research was conducted in accordance with the principles of the Declaration of Helsinki (1964) and subsequent revisions. All participants provided written informed consent for the anonymous use of their clinical and questionnaire data for research purposes.

## 3. Results

A total of 299 adult patients with psoriasis were included in the study. The majority were male (60%), and half of the participants fell within the age group of 50–69 years. Most patients had long-standing disease, with 52% reporting a disease duration of more than 10 years (Table 1).

In terms of disease severity, 61% of patients were classified as having moderate-to-severe psoriasis, based on clinical assessment. Despite this, a substantial portion of the population (58%) reported relatively low impairment in quality of life, as indicated by a DLQI score of less than 5. Only 12% of patients had a DLQI greater than 10, reflecting significant psychosocial and functional impact.

Therapeutic strategies were varied: 51% of patients were receiving topical treatment, phototherapy, or a combination of both, while 46% were undergoing systemic therapy. Among those on systemic treatment, 19% were on traditional agents such as methotrexate or cyclosporine, while 27% were treated with biologics. A small minority (3%) were not receiving any form of treatment at the time of evaluation.

Symptom analysis revealed a higher prevalence of subjective symptoms over the entire disease course compared to the four-week period immediately preceding the survey. Itch emerged as the most commonly reported symptom both historically and in the short term, with 89% of patients indicating that they had experienced itch at some point during their disease, and 73% reporting its presence in the previous four weeks. Burning sensation was the second-most frequently reported symptom (57% lifetime, 43% recent), followed by skin pain (47% lifetime, 27% recent). Among those reporting symptoms at the time of questionnaire administration, 63% had itch alone, 28% experienced itch and skin pain, and 18% reported itch and a burning sensation (Table 2).

Longer disease duration was significantly associated with increased frequency of itch (*p* = 0.033) and burning sensation (*p* = 0.019), while skin pain did not show a statistically significant correlation with disease duration. Itch, in particular, was consistently reported across all patient subgroups, highlighting its central role in the patient experience.

Treatment status had a notable impact on symptom reporting. Patients who were not receiving systemic therapy at the time of the survey reported significantly higher rates of itch (*p* = 0.001), skin pain (*p* = 0.034), and burning sensation (*p* = 0.019) compared to those under systemic treatment. Nevertheless, 91% of all participants reported some degree of symptom improvement following the initiation of any therapy, including both topical and systemic modalities, underscoring the importance of appropriate treatment in symptom control.

Objective measures of disease severity were positively correlated with symptom burden. Higher PASI and BSA scores were associated with an increased prevalence and intensity of itch, skin pain, and burning sensation. Specifically, itch demonstrated a statistically significant association with both the PASI (*p* = 0.045) and BSA (*p* = 0.001). Linear regression analysis further showed that, for every unit increase in the PASI, there was a corresponding 20% increase in itch VAS score, while each unit increase in the BSA was associated with a 7% increase in itch VAS.

Patients with higher DLQI scores also reported more frequent and intense symptoms. Among those with a DLQI greater than 10, 72% reported significant itch, which, in turn, was associated with psychological disturbances such as anxiety (41%), depression (17%), and impaired concentration at work (18%). Similar associations were noted for skin pain, suggesting a strong link between symptom severity and emotional well-being.

No statistically significant differences in symptom prevalence were found with regard to level of education or the presence of psoriatic arthritis (PsA). However, both itch and skin pain were slightly more common in patients with PsA, although these trends did not reach statistical significance.

## 4. Discussion

Despite the increasing awareness of psoriasis as a systemic inflammatory disease, symptoms like itch, burning sensation, and skin pain remain underestimated in clinical practice. These symptoms not only correlate with cutaneous inflammation but may also reflect a more complex interplay between immune dysregulation and neural mechanisms, suggesting a possible neuroimmune component underlying their manifestation [4,10]. Traditionally, the focus has been primarily directed toward visible skin lesions and objective measures such as the PASI and BSA. However, our study confirms that subjective symptoms, particularly pruritus, constitute a significant and prevalent burden for patients, regardless of the extent of visible disease.

Pruritus, in particular, emerged as the most frequently reported symptom in our cohort, consistent with the literature, where it is noted in up to 70% of psoriatic patients [7,8,9]. From a molecular and pathophysiological standpoint, itch can be driven by both internal and external triggers. Inflammatory cytokines such as IL-17 and IL-23 are directly involved in the modulation of the itch pathway by affecting sensory nerves and skin innervation [4,6,13]. Additionally, scratching due to pruritus itself perpetuates the inflammatory cycle. This is mediated by the release of neuropeptides such as substance P from nerve terminals, which act on C-fibers and promote the transmission of the itch signal to the central nervous system [7]. These neuropeptides also stimulate mast cells, further contributing to the inflammatory cascade [14].

Psychological stress appears to play an amplifying role. It induces the secretion of corticotropin-releasing hormone (CRH) from the pituitary gland, which, in turn, leads to mast cell degranulation [14]. The degranulated mast cells release histamine and other pruritogenic mediators, further sensitizing peripheral nerve endings. Moreover, the vasodilation observed in psoriatic plaques enhances local inflammation and facilitates the recruitment of immune cells, creating a positive feedback loop that worsens itch perception [15]. These findings not only support the immunologic basis of pruritus but also suggest its close relation with the neuroendocrine system and psychosocial factors.

Interestingly, similar trends have been observed in pediatric psoriasis. Caroppo et al. reported itch as the most common symptom in children affected by psoriasis, followed by burning sensation and skin pain [12]. This indicates that the burden of these symptoms is not age-dependent and should be considered relevant across all age groups.

Burning sensation and pain, although less frequently described, also contribute substantially to patient discomfort. These symptoms are believed to be the result of a combination of nociceptive and neuropathic mechanisms [4,16,17]. In particular, Patruno et al. explored the role of IL-33, a cytokine implicated in psoriasis, as a potential modulator of skin pain [8]. IL-33 belongs to the IL-1 family and acts as an alarmin released during tissue damage. Notably, it has been shown to trigger cutaneous hypernociception in mice [18]. This underscores a dual role of IL-33 in both inflammation and pain pathways.

The phenomenon of neurogenic inflammation also plays a pivotal role in the genesis of skin pain in psoriatic patients. It has been demonstrated that mast cells in the dermis of psoriatic plaques may exhibit reduced protease activity, which leads to the incomplete degradation of neuropeptides like substance P and vasoactive intestinal peptide (VIP) [5]. These substances then accumulate and bind to receptors on mast cells, promoting further degranulation and perpetuating the inflammatory state [10,19]. Such a cycle sustains the perception of pain, even when the visible extent of skin lesions is limited.

Importantly, our findings confirm that the intensity of these symptoms does not correlate linearly with PASI scores. Patients with mild-to-moderate psoriasis in terms of surface involvement can still experience severe pruritus or pain. This highlights the limitations of relying solely on objective metrics to assess disease burden. Previous studies have demonstrated that even a minimal BSA involvement of 1–2% can be associated with moderate-to-severe pain, especially when high-impact areas are involved (e.g., palms, soles, face, or genitalia) [20].

The underlying neuroimmune mechanisms that mediate both itch and pain include common pathways: substance P, calcitonin gene-related peptide (CGRP), mast cells, and unmyelinated C-fibers or thinly myelinated Aδ-fibers are all implicated in neurogenic inflammation, a hallmark of chronic psoriasis. These neural components sensitize the skin to both pruritic and painful stimuli, contributing to the complexity of the patient’s symptomatology. Korman et al. emphasized that even mild skin pain may be associated with increased rates of anxiety and depression [20].

Similarly, Misery et al., in a French nationwide study, found that patients reporting skin pain had significantly higher scores in both the DLQI and the physical and mental components of the SF-12 quality of life questionnaire [21].

From a therapeutic point of view, our findings underscore the need to move beyond lesion-centered assessment when selecting treatments. Biologics targeting IL-17 and IL-23 pathways have been shown to effectively reduce pruritus, supporting their immunological basis [8]. However, treatment options specifically designed to alleviate burning and skin pain are currently limited. This unmet need should guide future research and drug development efforts, especially considering that conventional topical therapies often fail to provide adequate relief for these symptoms.

Moreover, the inclusion of patient-reported outcomes (PROs) in clinical evaluations should be standardized and systematically applied. Current indices like the PASI and BSA fail to reflect the full spectrum of patient burden. Tools such as the DLQI, while including a single item addressing symptoms like itch and pain, are insufficient to capture their severity and impact. More comprehensive symptom-specific tools should be integrated into routine clinical practice to better align therapeutic choices with individual patient needs.

Then, it is clear that itch, burning sensation, and skin pain are prevalent and clinically significant symptoms in patients with psoriasis. They are often underrecognized yet critically important in determining quality of life. These findings support a paradigm shift toward patient-centered management of psoriasis, where symptom burden—not just lesion severity—guides therapeutic decisions. Future studies should aim to further elucidate the mechanisms behind these symptoms and evaluate targeted interventions, with the ultimate goal of improving overall patient care and outcomes in psoriasis.

### Key Results

1. Itch as the Dominant Symptom:

Itch was the most frequently reported symptom, present in 73% of patients over the previous 4 weeks and in 89% over the disease course. This confirms the existing literature and underlines the need for the systematic screening of pruritus in all psoriatic patients, regardless of disease stage or phenotype [4,7,10].

2. Impact of Treatment Modality on Symptoms:

Patients not receiving systemic therapy, particularly biologics, reported significantly higher rates of itch, pain, and burning (*p* < 0.05). This highlights the broader efficacy of systemic treatments not only in reducing visible lesions but also in controlling neurocutaneous symptoms [10,11].

3. Symptoms Not Proportional to PASI/BSA:

Notably, a considerable proportion of patients with low PASI and BSA scores still reported moderate-to-severe symptoms. This dissociation between clinical severity and subjective burden emphasizes the limitations of relying solely on the PASI and BSA to guide treatment decisions [4,5,6,7].

4. Strong Link Between Symptom Burden and Quality of Life:

Higher DLQI scores were significantly associated with the presence and intensity of all three symptoms. Among patients with DLQI > 10, 72% reported severe itch, and many reported associated psychological distress, including anxiety and depression. These findings support the integration of symptom burden into therapeutic endpoints and shared decision-making [20,22].

5. Correlation with Disease Duration and the PASI:

Longer disease duration was correlated with a higher frequency of itch and burning. Each unit increase in the PASI was associated with a 20% increase in itch VAS score, further supporting the inflammatory contribution to symptom intensity [10,11].

## 5. Conclusions

This study underscores the importance of systematically assessing skin-related symptoms such as itch, burning sensation, and pain in patients affected by psoriasis. While, historically, these symptoms have been considered secondary to the visible skin lesions, emerging literature and our findings suggest that they play a fundamental role in the patient’s disease experience, influencing both psychological well-being and quality of life.

Pruritus is the most frequently reported symptom, yet often remains underrecognized during clinical assessments. Its multifactorial origin, which involves immune dysregulation, neurogenic inflammation, and psychological stress, highlights the complexity of symptom perception in psoriatic individuals. Moreover, skin pain and a burning sensation, though less studied, are not rare and may stem from both nociceptive and neuropathic pathways.

What emerges from our analysis is that the presence and severity of skin symptoms are not always proportional to objective clinical measures such as the PASI and BSA. Patients with a low lesion extent may still report intense pruritus or pain, and this discrepancy emphasizes the need for a more patient-centered approach. In line with modern dermatological practice, which values not only the clinician’s objective assessment but also the patient’s subjective experience, it becomes evident that symptom burden must be integrated into the therapeutic decision-making process.

The Dermatology Life Quality Index (DLQI), though widely used, includes only one item dedicated to skin symptoms. This may lead to an underestimation of their impact, especially in those patients who do not present with extensive disease but suffer from intense discomfort. A more granular approach to symptom tracking—potentially via Visual Analog Scales or Numeric Rating Scales—should be encouraged in daily practice.

From a therapeutic standpoint, while current biologic treatments targeting the IL-23/IL-17 axis show efficacy in reducing both inflammation and itch, specific data on their effects on burning and pain remain limited. This points to an unmet clinical need: the development of therapeutic strategies aimed not only at achieving complete skin clearance but also at controlling neurogenic inflammation and restoring the skin’s sensory balance. Such a shift would mark an evolution from lesion-focused management toward a more holistic vision of care.

Further research is needed to fully elucidate the mechanisms underlying skin symptoms in psoriasis and to identify biomarkers that may help predict their severity or response to therapy.

In conclusion, itch, pain, and a burning sensation are not marginal manifestations of psoriasis, they are central components of the disease burden. Their recognition should be systematic, and their treatment prioritized for fostering a truly patient-oriented model of care.

## Figures and Tables

**Table 1:** Demographic characteristics of the patients enrolled in the study.

Characteristics	%
Sex	
Male	60
Female	40
Age (years)	
<40	20
40–49	16
50–59	24
60–69	24
>70	16
Education	
Elementary school	9
Middle school	35
High school	40
University	17
Psoriatric Arthritis	29
Disease duration	
<10 years	48
>10 years	52
Ongoing therapy	
Topical	18
Phototherapy	20
Topical + phototherapy	13
Traditional systemic	19
Biologic	27
None	3

**Table 2:** Percentage of patients reporting itch, pain, or a burning sensation 4 weeks before the questionnaires were administered. * Test compared with 0.05, as statistically significative.

	Itch		Pain		Burning	
	%	*p* Value	%	*p* Value	%	*p* Value
**SEX**		0.247		0.993		0.121
M	70.2		27.1		40.9	
F	76.3		27.1		50	
**AGE**		0.606		0.887		0.265
<40	69.5		30.5		54.2	
40–49	72.2		25		47.9	
50–59	70.9		29.6		46.5	
60–69	75.1		26		37	
>70	77.1		22.9		37.5	
**DISEASE DURATION**		0.033 *		0.103		0.029 *
<10 years	78.3		31.5		51	
>10 years	67.3		23.1		38.5	
**EDUCATION LEVEL**		0.107		0.223		0.814
Elementary	88.9		29.6		37	
Middle School	68		30		45	
Diploma	70		29.2		46.7	
University	78.8		15.4		42.3	
**ARTHRITIS**		0.781		0.219		0.925
Yes	71.4		32.1		44	
No	73		25.1		44.7	
**TREATMENT**		<0.001 *		0.034 *		0.019 *
Topical	89.1		43.6		60	
Phototherapy	84.7		22		42.4	
Topical + Phototherapy	73.7		31.6		50	
Systemic	72.4		22.4		39.7	
Biological	50.6		19.8		33.3	
No	87.5		37.5		75	
**PASI**		0.045 *		0.763		0.099
<5	71.1		26.3		52.6	
5–7	82.7		32.7		46.2	
8–10	62.8		24.4		37.2	
>10	80		27.3		36.4	
**BSA**		0.001 *		0.117		0.069
<5	68.7		29.3		49.5	
5–9	83.3		25.8		51.5	
10–14	80.8		23.5		33.8	
15–19	96.2		46.2		53.8	
>20	72.5		17.5		36.4	
**DLQI**		0.709		0.697		0.075
0–1	69.5		30.5		54.2	
1–5	76.3		27.2		48.2	
6–10	70		27.8		38.9	
>11	72.2		19.4		30.6	

## Data Availability

The raw data supporting the conclusions of this article will be made available by the authors on request.

## Supplementary Materials

The following supporting information can be downloaded at https://www.mdpi.com/article/10.3390/jcm14134388/s1: Table S1: Itch questionnaire administered to patients; Table S2: Burning sensation questionnaire administered to patients; Table S3: Pain questionnaire administered to patients.

## Abbreviations

The following abbreviations are used in this manuscript:

PASI	Psoriasis Area and Severy Index
DLQI	Dermatology Life Quality Index
BSA	Body Surface Area
IL	Interleukin
TNF	Tumor Necrosis Factor
NGF	Nerve Growth Factor
VAS	Visual Analogic Scale
NRS	Numeric Rating Scale
PROMs	Patient-Reported Outcome Measures
PsA	Psoriatic Arthritis
SF-12 PCS	Short Form-12 Physical Composite Score
SF-12 MCS	Short Form-12 Mental Composite Score
CRH	Corticotropin Releasing Hormone
VIP	Vasoactive Intestinal Peptide
CGRP	Calcitonin Gene-Related Peptide
