# Supplementary material for: Cutaneous Psoriasis and Symptoms (Itch, Pain, and Burning Sensation): A Monocentric Retrospective Study on 299 Patients in Italy
Source: J Clin Med. 2025 Jun 20;14(13):4388. doi: 10.3390/jcm14134388 (PMC12249676; doi:10.3390/jcm14134388)
Supplement: Supplementary file 1 [file jcm-14-04388-s001.zip › Table S1 Itch questionnaire administered to patients.pdf]

1) Have you ever suffered from itch? Yes□ No□

2) Scratching modifies itch Yes□ No□
If yes, how?
It improves □
It worsen □

3) Does the ongoing therapy improve itch? Yes□ No□

4) Did you experience itch in the last 4 weeks? Yes□ No□

5) Could you indicate the intensity of your itch (0 = none; 10= highest) in the last 4 weeks

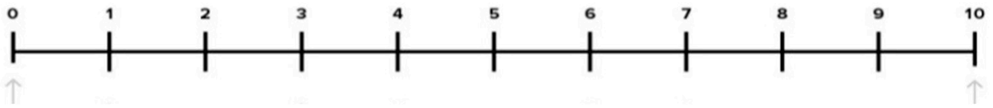

6) Does itch have any impact on your life? Yes□ No□
If yes, how?
I feel depressed □
I feel anxious □
I have trouble stay focused □
I changed my dietary habits □
My sexual life is impaired □

6) Did you notice differences of itch during the day/the year? Yes□ No□
If yes, how?
It worsen in the morning □
It worsen during the day □
It worsen at night □
It worsen in spring □
It worsen in summer □
It worsen in autumn □
It worsen in winter □

7) Is it itch associated with other symptoms? Yes□ No□
If yes, which?
Skin pain □
Burning sensation □
Both □
