# Supplementary material for: Cutaneous Psoriasis and Symptoms (Itch, Pain, and Burning Sensation): A Monocentric Retrospective Study on 299 Patients in Italy
Source: J Clin Med. 2025 Jun 20;14(13):4388. doi: 10.3390/jcm14134388 (PMC12249676; doi:10.3390/jcm14134388)

1) Have you ever suffered from burning sensation? Yes□   No□

2) Scratching modifies burning sensation   Yes□   No□
If yes, how?
It improves   □
It worsen   □

3) Does the ongoing therapy improve burning sensation? Yes□   No□

4) Did you experience burning sensation in the last 4 weeks? Yes□   No□

5) Could you indicate the intensity of your burning sensation (0 = none; 10= highest) in the last 4 weeks

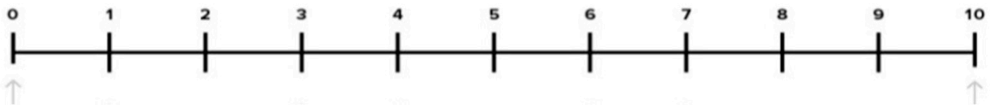

Supplement: Supplementary file 1 [file jcm-14-04388-s001.zip › Table S2 Burning sensation questionnaire administered to patients.pdf]
